# Effect of Silver Diamine Fluoride and Potassium Iodide Treatment on Secondary Caries Prevention and Tooth Discolouration in Cervical Glass Ionomer Cement Restoration

**DOI:** 10.3390/ijms18020340

**Published:** 2017-02-06

**Authors:** Irene Shuping Zhao, May Lei Mei, Michael F. Burrow, Edward Chin-Man Lo, Chun-Hung Chu

**Affiliations:** 1 Faculty of Dentistry, The University of Hong Kong, Hong Kong 999077, China; zhao110@hku.hk (I.S.Z.); mei1123@hku.hk (M.L.M.); hrdplcm@hku.hk (E.C.-M.L.); 2 Melbourne Dental School, University of Melbourne, Melbourne 3010, Australia; mfburrow@unimelb.edu.au

**Keywords:** silver diamine fluoride, potassium iodide, secondary caries, glass ionomer, discolouration

## Abstract

This study investigated the effect of silver diamine fluoride (SDF) and potassium iodide (KI) treatment on secondary caries prevention and tooth discolouration in glass ionomer cement (GIC) restoration. Cervical GIC restorations were done on 30 premolars with: Group 1, SDF + KI; Group 2, SDF (positive control); Group 3, no treatment (negative control). After cariogenic biofilm challenge, the demineralisation of dentine adjacent to the restoration was evaluated using micro-computed tomography (micro-CT) and Fourier transform infrared (FTIR) spectroscopy. The colour of dentine adjacent to the restoration was assessed using CIELAB system at different time points. Total colour change (∆E) was calculated and was visible if ∆E > 3.7. Micro-CT showed the outer lesion depths for Groups 1, 2 and 3 were 91 ± 7 µm, 80 ± 7 µm and 119 ± 8 µm, respectively (*p* < 0.001; Group 2 < Group 1 < Group 3). FTIR found that there was a significant difference in amide I-to-hydrogen phosphate ratio among the three groups (*p* < 0.001; Group 2 < Group 1 < Group 3). ∆E of Groups 1, 2 and 3 after biofilm challenge were 22.5 ± 4.9, 70.2 ± 8.3 and 2.9 ± 0.9, respectively (*p* < 0.001; Group 3 < Group 1 < Group 2). SDF + KI treatment reduced secondary caries formation on GIC restoration, but it was not as effective as SDF treatment alone. Moreover, a perceptible staining on the restoration margin was observed, but the intensity of discolouration was less than that with solely SDF treatment.

## 1. Introduction

Secondary (recurrent) caries, which refers to the carious lesions affecting the margins of an existing restoration [1], is regarded as the most common reason for re-restoration of teeth in the long term [2]. It has been reported that more than 25% of restoration replacements of amalgam and resin composite were ascribed to secondary caries [3]. This fact has facilitated the development of dental materials that possess anti-cariogenic properties, such as fluoride-containing restorative materials [4]. Glass ionomer cements (GICs) can release fluoride ions to enhance remineralisation, and their abilities in fluoride release and recharge are superior to other restorative materials, such as compomers and giomers [4]. However, its antimicrobial effect is limited and inadequate to prevent secondary caries development [5].

The cariogenic bacteria of secondary caries are similar to those of primary caries, and consist primarily of *Streptococci*, *Actinomyces naeslundii* and *Lactobacilli* [2]. Studies have shown that silver diamine fluoride (SDF) has an intense antibacterial effect on cariogenic bacteria and can inhibit the growth of multi-species cariogenic biofilms on tooth surfaces [6,7,8]. SDF is a topical fluoride which is often used in high concentration (38%) for preventing and arresting dental caries [9]. SDF has recently been approved for clinical use by the United States Food and Drug Administration in 2015. A review concluded SDF as an effective, efficient, equitable and safe caries-preventive agent appearing to meet the World Health Organization’s Millennium Goals for 21st century medical care [10]. Clinical studies also showed the success of SDF in preventing and arresting dental caries [11,12]. A laboratory study found that the bond strength of restorations to dentine was not adversely affected by SDF using resin-based adhesives [13]. The application of SDF under GIC restorations has been demonstrated to produce a promising pulpal response and be effective in facilitating the formation of reparative dentine [14]. It also has been reported that prior treatment with SDF can increase resistance of cavity margins around GIC restorations to secondary caries development [5].

A significant disadvantage of SDF use, however, is black staining on teeth which can cause aesthetic concern [11]. A way that has been suggested of managing this problem is to apply a saturated solution of potassium iodide (KI) immediately after SDF application. It was suggested that discolouration of the carious lesion can be avoided while the caries arresting effect of SDF is not changed [15]. The suggested explanation is that the silver ions from the SDF solution will react with the iodide ions from the KI solution to form silver iodide. It was reported that the application of SDF + KI to dentine surfaces before the placement of GIC restorations did not affect the bond strength of GIC to dentine [16], and did not adversely interfere with the fluoride uptake into the adjacent demineralised dentine [15]. It would be desirable if KI could inhibit the staining formation associated with SDF without diminishing its effectiveness in preventing and arresting dental caries. Nevertheless, evidence from laboratory data is insufficient to support this claim. A search in PubMed found that there was no study reporting the effect of SDF + KI treatment in the prevention of secondary caries formation on GIC restorations, and quantifying the discolouration of tooth structure after the application of SDF + KI. Therefore, the objectives of this laboratory study were to investigate the effect of SDF + KI treatment on the prevention of secondary root caries development around direct GIC restorations, and to assess whether SDF + KI treatment could prevent discolouration of the dentine adjacent to GIC restorations. The first null hypothesis was that SDF + KI treatment has no effect on secondary caries prevention around GIC restorations. The second null hypothesis was that SDF + KI treatment has no staining effect on dentine along the restoration margin.

## 2. Results

**Figure 1:**
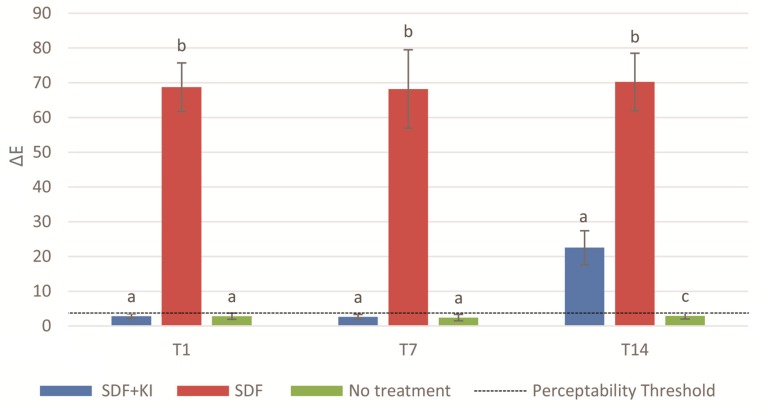
Total colour change (∆E) of the three groups. T1: after material filling (after setting for 1 day), T7: after thermal-cycling (7 days after material placement), and T14: after biofilm challenge (14 days after material placement). Columns displaying different letters indicate a significant difference (*p* < 0.05) between groups within each time point.

Results of one-way ANOVA showed that the colour of the three groups was not significantly different at the baseline L* (*p* = 0.974), a* (*p* = 0.920) and b* (*p* = 0.352). L* axis represented lightness ranged from black (0) to white (100), a* axis described red (+a*) to green (−a*), and the b* axis represented yellow (+b*) to blue (−b*). The values relating to the chromatic coordinates L* a* b* and total colour change ∆E of the three groups are presented in Table 1 and Figure 1, respectively. For the intragroup analysis, there was a significant drop in the values of lightness (L*) in Group 1 from T (time point) 7 to T14 (*p* < 0.001), while Group 2 displayed a significant decrease in L* values from T0 to T1 (*p* < 0.001) (Table 1). The negative control did not show a noticeable colour variation at any time point (∆E < 3.7, *p* < 0.05) (Figure 1). Group 1 displayed a perceptible colour difference of ∆E > 3.7 at T14, whereas Group 2 presented a perceptible colour change from T1 onward (Figure 1). Pair-wise comparisons revealed that Group 1 (∆E = 22.5 ± 4.9) exhibited a colour difference inferior to that of Group 2 (∆E = 70.2 ± 8.3) at T14 (*p* < 0.001).

**Figure 2:**
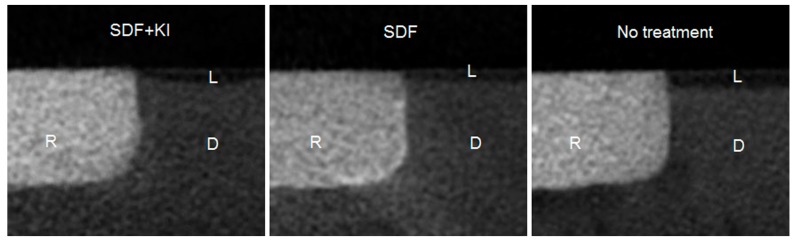
Micro-computed tomography (micro-CT) images of the three groups. SDF + KI: Group 1, the cavity was treated with silver diamine fluoride and potassium iodide; SDF: Group 2, the cavity was treated with silver diamine fluoride as positive control; No treatment: Group 3, negative control; R: glass ionomer cement (GIC) restoration; D: dentine; L: demineralised outer lesion.

**Figure 3:**
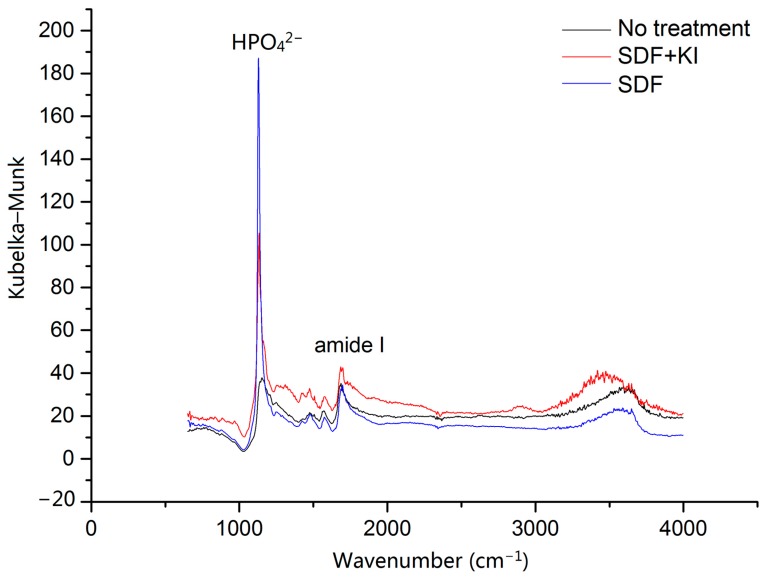
Fourier transform infrared (FTIR) spectra of dentine at the material-root junction. SDF + KI: Group 1, the cavity was treated with silver diamine fluoride and potassium iodide; SDF: Group 2, the cavity was treated with silver diamine fluoride as positive control; No treatment: Group 3, negative control. The strongest peak of HPO_4_^2−^ was found in Group 2, followed by Group 1 and 3.

Representative images of micro-computed tomography (micro-CT) and Fourier transform infrared (FTIR) spectra from the three groups are displayed in Figure 2 and Figure 3, respectively. The mean outer lesion depth (±SD) of Groups 1, 2 and 3 was 91 ± 7 µm, 80 ± 7 µm and 119 ± 8 µm, respectively (*p* < 0.001; Group 2 < Group 1 < Group 3). The values of the amide I: HPO_4_^2−^ absorbance ratio of Groups 1, 2 and 3 were 0.50 ± 0.05, 0.43 ± 0.03 and 0.71 ± 0.05, respectively (*p* < 0.001; Group 2 < Group 1 < Group 3).

## 3. Discussion

SDF at a concentration of 38% was chosen as the positive control, because it is well known that it is effective in preventing and arresting dental caries [10,17]. Laboratory studies have illustrated that the topical application of a 38% SDF solution can inhibit the growth of cariogenic biofilms [6,7,8]. It has been suggested that 38% SDF possesses a strong inhibitory effect on the action of cysteine cathepsins [18] and matrix metalloproteinase [19], which are closely related to the collagen degradation of dentine. In addition, SDF treatment can increase the micro-hardness of carious lesions in dentine [20] and the mineral density of carious lesions in enamel [21]. Clinical studies have shown that 38% SDF arrested coronal caries in children [11] and prevented root caries in elderly patients [22]. However, SDF can cause black staining of tooth structure which may not be acceptable to many patients from the aesthetic point of view. A promising approach to solve this problem is to apply the KI solution immediately after SDF treatment. To make this study more relevant to clinicians, SDF products readily available on the market were used. Both Saforide and Riva Star contain 38% SDF and are commercially available. Saforide was selected as positive control because it is the most common 38% SDF product used in previous studies. The only commercial product of 38% SDF + KI available is Riva Star. Hence, it was used in the experimental group.

There are various techniques available for disinfection of extracted teeth, for instance, γ irradiation, ethylene oxide, and autoclaving. γ Irradiation can sterilise the teeth without altering the tooth structure and the function of dentine. However, it requires expensive equipment, which is not easily accessible [23]. It is believed that surface crazing and cracks were likely to have developed in the GIC restorative material if sterilisation with ethylene oxide was adopted as a result of dehydration. Therefore, autoclaving was used to sterilise the teeth in this study. Autoclaving is an effective, cheap, simple and chemically safe method suitable for tooth sterilisation [24], which was been recommended in previous laboratory studies [5,25]. Nevertheless, there was some concern with regard to the high pressure and temperature required which may damage the dentinal structure or denature the collagen. Although autoclaving of teeth may reduce the micro-hardness of dentine, the reduction in micro-hardness is minimal and it does not affect the physical properties to the degree of compromising strength [23]. Moreover, the dentine collagen structure can be weakened by autoclaving, but it would not be destroyed to any major degree, because the molecular structure of dentine collagen has been reported to remain relatively unaffected [25].

Secondary caries development has been commonly reported in two locations: along the cavity wall adjacent to the restoration (namely wall lesion) [1], but also at the tooth surface next to the filling material (namely outer lesion), similar to primary caries. The depth of these two kinds of lesions has been commonly used to assess the inhibitory effect on the development of secondary caries [5]. An in situ study found that wall lesions only formed when there was a gap between the restoration and the tooth, indicating that the presence of a gap is a crucial condition for the development of a wall lesion [1]. Another laboratory study reported that wall lesions were only detected in the resin composite group but not in the GIC group, and the possible explanation was that the shrinkage of resin composites could cause contraction away from the cavity walls [5]. However, some researchers have the concern that the “wall lesion” has been used indiscriminately, and it is uncertain whether an entity like a wall lesion exists per se clinically [2]. Thus, the assessment of wall lesion depth was not adopted in the current study. Moreover, this study did not find any recognisable wall lesion in all specimens (data not shown). The reason could be there was no gap between the restorations and the teeth, and the use of GIC which released fluoride and promoted remineralisation.

FTIR is an easy approach to identify the existence of molecular functional groups (namely, HPO_4_^2−^ and amide I) and thus was adopted in the current study. However, the instrument should be repetitively calibrated, since the analog connection between the recording device and the monochromater position is prone to misalignment and wear. The mineral of dentine is composed of hydroxyapatite, and the organic matrix fraction is mainly composed of type I collagen. The HPO_4_^2−^ band in the FTIR spectrum is representative of the mineral, while the amide I band represents the secondary structure of collagen [26]. The ratio of amide I: HPO_4_^2−^ indicates the extent of demineralisation of dentine. In this study, the amide I: HPO_4_^2−^ ratio was lowest in group SDF, followed by group SDF + KI and negative control. This result demonstrated that SDF with or without KI, inhibited demineralisation of dentine and prevented secondary caries formation.

Previous laboratory studies reported that both SDF solution and SDF + KI solution inhibited cariogenic biofilm formation on demineralised dentine [27,28]. Moreover, the studies also found SDF and SDF + KI could reduce the permeability of cariogenic bacteria through demineralised dentine slices, and increase the resistance to further demineralization without significant differences between these two treatments [27,28]. In this study, SDF + KI solution could prevent secondary caries formation around GIC restorations, but it was not as effective as SDF. This finding might suggest KI may influence the effectiveness of SDF in preventing the formation of secondary root caries. The probable reason is that the application of KI solution might reduce the amount of silver ions. It is known that silver ions contained in the SDF solution play an important role in antimicrobial activities to hinder caries progression.

The aesthetic appearance of a restoration is an important concern of patients. Rather than assessing colour differences by the naked eye which is often subjective, our evaluation was to quantify colour changes using instrument-based measurements which are more precise with a high repeatability [29]. Silver ions in the SDF solution can blacken the tooth structure. It is suggested that the KI solution can react with SDF to form a bright yellow solid compound (silver iodide) [30], and this reaction could reduce the excess free silver ions which result in the black staining [27]. Although the bright yellow precipitates can be seen after the application of KI, the staining of tooth surfaces could still be detected in SDF + KI treatment group in this study. While KI was supposed to remove the staining caused by SDF, its effect has not been previously quantified. In this study, SDF + KI treatment led to discolouration of tooth surfaces although the intensity of the discolouration was less than that of SDF treatment. One possible explanation may be that the amount of the applied KI solution was not sufficient to lead to an excess of free silver ions remaining [30]. Besides, silver iodide is considered to be highly photosensitive which can dissociate into metallic silver and iodine by exposure to light. Hence, discolouration still occurred on tooth surfaces.

To our knowledge, this study is the first laboratory study to investigate the effectiveness of SDF + KI treatment in secondary root caries prevention around GIC restorations, and to quantify the discolouration on the restoration margins caused by SDF + KI. Based on the results of this study, the two null hypotheses were rejected. The results demonstrated that SDF + KI treatment could increase resistance of GIC restorations to the formation of secondary root caries, but was not as effective as SDF treatment. Moreover, in the long term, it was not effective in preventing discolouration of the restoration margin, but could reduce staining compared to that of SDF. It is noteworthy that this laboratory study is based on a laboratory model, which is different from the more complex clinical situation. The results cannot be extrapolated directly to the in vivo condition and caution is advised in the interpretation of the results.

## 4. Materials and Methods

### 4.1. Specimen Preparation and Materials Selection

The use of human teeth in this study was approved by the Institutional Review Board of the University of Hong Kong/Hospital Authority Hong Kong West Cluster (IRB UW 12-221). Thirty extracted sound human upper and lower premolars from 10 patients were collected with the patients’ consent. The premolars were stored in a 0.1% thymol solution at 4 °C before use and were used within 1 month of extraction. Box-shaped cavities (4 × 2 × 2 mm^3^) along the cemento-enamel junction were prepared on premolars. The cavities were prepared with a tungsten carbide bur (FG 330; SS White, Lakewood, NJ, USA) under copious water cooling. Then the teeth were sterilised by autoclaving at 121 °C [25]. The cavities were conditioned with 10% polyacrylic acid [5] and randomly allocated to the following three treatment groups:

- Group 1 (SDF + KI), the cavity was treated with SDF + KI (Riva Star, SDI, Bayswater, Australia). A layer of 38% SDF solution was topically applied to the cavity, immediately followed by a saturated KI solution until the creamy white solution turned clear. The reaction products were washed off with copious distilled water [28]. Then the cavity was dried with oil-free compressed air and filled with GIC (Fuji VII capsule, GC International, Tokyo, Japan).
- Group 2 (SDF), positive control—the cavity was treated with 38% SDF (Saforide; Toyo Seiyaku Kasei Co., Ltd., Osaka, Japan) for 3 min, followed by GIC restoration (Fuji VII capsule).
- Group 3 (no treatment), negative control, the cavity was filled with GIC (Fuji VII capsule).

**Figure 4:**
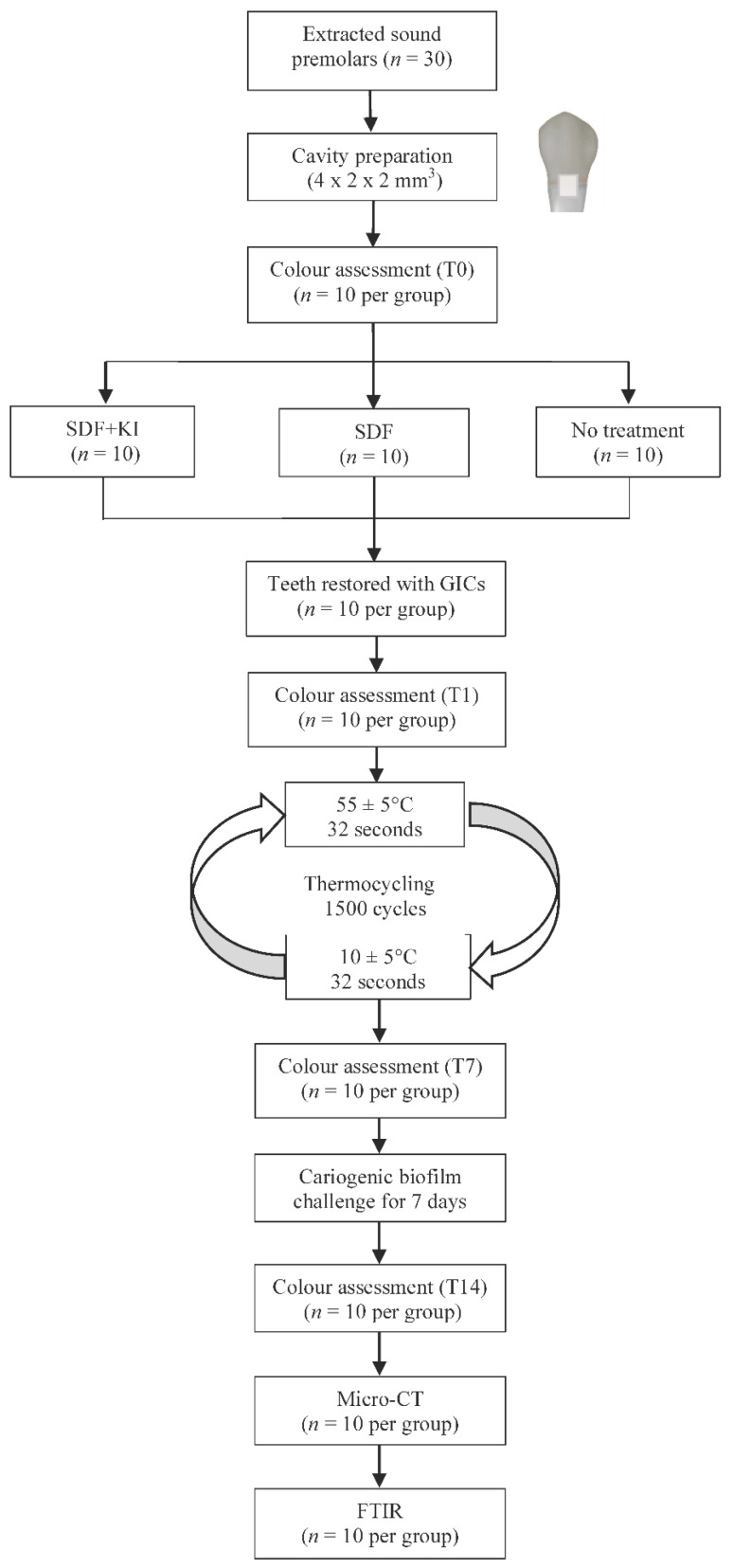
Flow chart of the study.

The flow chart of the present study is shown in Figure 4. All procedures were performed with sterile instruments and gloves. The restored teeth were then stored at 37 °C and 100% humidity for 24 h. The restoration surfaces were finished and polished using 4000 grit sanding paper to confirm there was no excess over the cavity margins. To simulate the aging process, the restored teeth were thermocycled for 1500 cycles in 55 ± 5 °C and 10 ± 5 °C distilled water baths with a 32 s dwell time in each bath and a 14 s interval between baths [5]. Then the teeth were immersed in 70% alcohol for 60 s and air dried for 20 s before undergoing a cariogenic biofilm challenge [31].

### 4.2. Cariogenic Biofilm Challenge

Cariogenic bacteria used for biofilm challenge were *Streptococcus mutans* ATCC 35668 (American Type Culture Collection), *Streptococcus sobrinus* ATCC 33478, *Lactobacillus rhamnosus* ATCC 10863 and *Actinomyces naselundii* ATCC 12014 [26]. The microorganisms were cultured on blood agar plates for 2 days (37 °C, anaerobically). Then, a single colony was picked from each agar plate and transferred to tubes containing brain-heart infusion (BHI) broth with 5% sucrose to prepare 24 h broth cultures at 37 °C under anaerobic conditions. After that, the bacterial cell pellets were harvested by centrifugation (1500× *g*, 37 °C, 10 min). Bacterial suspensions were then prepared in BHI broth with 5% sucrose to a cell density of McFarland 2 (6 × 10^8^ cells/mL) [5]. Each restored tooth was soaked in a well of a 12-well plate containing 500 µL of each bacteria culture. The plate was maintained in an anaerobic chamber at 37 °C for 7 days. The medium was refreshed daily [7] and Gram stain test of the used medium was performed to check contaminants.

### 4.3. Colour Assessment

Colour assessments (*n* = 10 per group) were taken at four time points: T0: baseline (after preparation of the cavities), T1: after material filling (after setting for 1 day), T7: after thermal-cycling (7 days after material placement), and T14: after biofilm challenge (14 days after material placement). The colour of the dentine surface adjacent to the restoration was observed using a VITA Easyshade^®^ advanced portable dental spectrophotometer (VITA Zahnfabrik GmbH, Bad Säckingen, Germany). Each colour was elucidated three-dimensionally in space according to the Commission International del’Eclairage L* a* b* colour system. L* axis represented lightness ranged from black (0) to white (100), a* axis described red (+a*) to green (−a*), and the b* axis represented yellow (+b*) to blue (−b*). The instrument was calibrated with the manufacturer’s instruction before examination. The L*, a* and b* values were replicated three times by a single operator and the average values were recorded. The difference of colour between baseline and each time point was calculated based on the mathematical equation ∆E = [(∆L)^2^ + (∆a)^2^ + (∆b)^2^]^1/2^ [29]. The perceptibility threshold of ∆E, which the tooth colour change was clinically visible to the naked eye, was set at 3.7 units [32].

### 4.4. Outer Lesion Depth Assessment

The assessing method for outer lesion depth was adapted from Mei et al. [5]. The teeth (*n* = 10 per group) were scanned non-destructively in container tubes with water [33] using a SkyScan 1076 micro-CT (SkyScan, Antwerp, Belgium) to measure the outer lesion depth. The spatial resolution of 8 µm was used for scanning. The X-ray source was operated at 80 kV and 100 µA. A 1 mm thick aluminium filter was employed in front of the detector to eliminate low-energy radiation. Scanning results were reconstructed by the reconstruction software NRecon (SkyScan). Afterwards, reconstructed images were viewed by the data analysing software CTAn (SkyScan). From the reconstructed three-dimensional images, cross-sectional images of each tooth exhibiting lesion area were identified. Ten images were chosen by random sampling from those lesion images [34]. The outer lesion depth was quantified using ImageJ software [35].

### 4.5. Structural Evaluation of Dentine

The analysis of potential changes in the organic structure of restoration margins were performed by FTIR spectroscopy (UMA 500, Bio-Rad Laboratories, Hercules, CA, USA) with the infrared radiation ranging from 650 to 4000 cm^−1^ in wavelength number [8]. Spectra of the demineralised dentine adjacent to the restoration (*n* = 10 per group) were obtained by the average acquisition of data at the spatial resolution achieved with a 50 × 50 µm aperture. The ratio of the integrated area of collagen amide I absorbance between 1585 and 1720 cm^−1^ to that of HPO_4_^2−^ absorbance between 900 and 1200 cm^−1^ was calculated. The value of the amide I: HPO_4_^2−^ absorbance ratio indicated the extent of demineralisation of root dentine as a result of the activity of the cariogenic biofilm [8].

### 4.6. Statistical Analysis

All data were assessed for normality using the Shapiro–Wilk test (*p* > 0.05). Repeated measures analysis of variance (ANOVA) was applied to evaluate L*, a* and b* values over time within each group. One-way ANOVA with Bonferroni post hoc test was used to detect differences in ∆E (at different time points), outer lesion depth and amide I: HPO_4_^2−^ between groups. One-sample Student’s *t*-test was used for each group at different time points to test whether the color change significantly different from the standard as 3.7. All analyses were conducted using IBM SPSS Version 20.0 software (IBM Corp., Armonk, NY, USA). The level of significance was set at 5%.

## 5. Conclusions

SDF + KI treatment inhibited development of secondary caries on GIC restorations, but was not as effective as SDF treatment alone. Moreover, SDF + KI treatment caused a perceptible staining at the restoration margin, but the intensity was less than that with purely SDF treatment.

## Figures and Tables

**Table 1:** Mean (±SD) values of L* a* b* coordinates of the three groups (*n* = 10).

Group	Coordinates	T0	T1	T7	T14	*p*-Value
SDF + KI	L*	90.2 ± 6.0	89.9 ± 5.1	88.6 ± 6.4	68.5 ± 4.2	<0.001
	a*	0.6 ± 0.9	0.6 ± 0.9	0.6 ± 0.7	4.2 ± 1.1	<0.001
	b*	33.6 ± 4.7	33.4 ± 5.2	33.9 ± 3.1	34.0 ± 4.3	0.840
SDF	L*	89.5 ± 6.9	25.3 ± 4.1	27.4 ± 8.1	24.7 ± 5.8	<0.001
	a*	0.6 ± 1.0	4.5 ± 0.9	3.7 ± 1.6	3.9 ± 1.3	<0.001
	b*	35.9 ± 5.1	12.2 ± 1.9	8.5 ± 4.4	9.6 ± 3.3	<0.001
No treatment	L*	89.2 ± 5.2	88.4 ± 6.0	88.4 ± 6.1	88.0 ± 5.5	0.281
	a*	0.7 ± 1.1	0.6 ± 1.0	0.7 ± 0.9	0.6 ± 1.0	0.951
	b*	32.8 ± 4.7	33.2 ± 4.1	32.5 ± 4.9	33.1 ± 4.1	0.702

T0: baseline (after preparation of the cavities), T1: after material filling (after setting for 1 day), T7: after thermal-cycling (7 days after material placement), and T14: after biofilm challenge (14 days after material placement). L* axis represented lightness ranged from black (0) to white (100), a* axis described red (+a*) to green (−a*), and the b* axis represented yellow (+b*) to blue (−b*). SDF: silver diamine fluoride; KI: potassium iodide.

## Abbreviations

SDF	Silver diamine fluoride
KI	Potassium iodide
GIC	Glass ionomer cement
Micro-CT	Micro-computed tomography
FTIR	Fourier transform infrared
